# The first global multi-timescale daily SPEI dataset from 1982 to 2021

**DOI:** 10.1038/s41597-024-03047-z

**Published:** 2024-02-21

**Authors:** Xuebang Liu, Shuying Yu, Zhiwei Yang, Jianquan Dong, Jian Peng

**Affiliations:** Laboratory for Earth Surface Processes, Ministry of Education, College of Urban and Environmental Sciences, Peking University, Beijing, 100871 China

**Keywords:** Hydrology, Natural hazards

## Abstract

Global warming accelerates water cycle, causing more droughts globally that challenge monitoring and forecasting. The Standardized Precipitation Evapotranspiration Index (SPEI) is used to assess drought characteristics and response time of natural and economic systems at various timescales. However, existing SPEI datasets have coarse spatial or temporal resolution or limited spatial extent, restricting their ability to accurately identify the start or end dates or the extent of drought at the global scale. To narrow these gaps, we developed a global daily SPEI dataset (SPEI-GD), with a 0.25° spatial resolution from 1982 to 2021 at multiple timescales (5, 30, 90, 180 and 360 days), based on the precipitation from European Center for Medium Weather Forecasting Reanalysis V5 (ERA5) dataset and the potential evapotranspiration from Singer’s dataset. Compared to widely used SPEIbase dataset, the SPEI-GD can improve the spatial-temporal resolution and the accuracy of SPEI in areas where meteorological sites are lacking. The SPEI-GD significantly correlates with site-based SPEI and soil moisture. Our dataset solidly supports sub-seasonal and daily-scale global and regional drought research.

## Background & Summary

Drought is a major natural hazard caused by a persistent water deficit over a period of time^1^, which can cause devastating impacts on regional agriculture^2–4^, water resources^5^ and vegetation coverage^6,7^, as well as on human health^8^, with far-reaching influences in an increasing globalized world^9^. For example, during 2003 central Europe drought, the gross primary productivity was estimated to reduce by 30%, which was equal to 4-year net carbon uptake of European ecosystem^10^. A more severe drought attacked Russia in 2010, which caused ~55,000 deaths, reduced crop yields by ~25%, and led to ~US$15 billion total economic loss^5,11^. As global warming accelerates the terrestrial water cycle^12^, droughts have increased substantially in many regions^13,14^ and are projected to become more frequent, severer and longer in the warmer future^1,15^. Therefore, deeply understanding and monitoring drought is crucial to carry out risk management and adaptive strategy for drought hazard.

Droughts are usually classified into four categories: meteorological, agricultural, hydrological, and socioeconomic drought^16^. Propagation can occur in different droughts. That is to say, the lack of precipitation accompanying meteorological drought can lead to the deficiency of soil moisture, runoff or regional water availability, implying that meteorological drought can propagate into agricultural, hydrological or socioeconomic drought^17–19^. Meanwhile, different types of drought have differed characteristics, in terms of intensity, duration and frequency, making it very difficult to characterize quantitatively^20,21^. In addition, different physical mechanisms can cause drought at different timescales. For droughts at interannual or decadal timescales, large-scale internal climate variability (e.g. El Niño–Southern Oscillation or Pacific Decadal Variability) play critical roles^22,23^. For droughts at seasonal timescale, the local and remote land-atmosphere feedbacks are dominant drivers^24,25^. For droughts at sub-seasonal timescales or rapid-onset drought (flash drought), which can develop into severe droughts within a few weeks, the primary drivers are large precipitation deficits and abnormally high evapotranspiration^26,27^. These various and multiscale droughts can interact among above climate drivers to produce complex drought characteristics, raising great challenges for drought forecasting and impact mitigation^28^.

In order to accurately assess droughts, several drought indices have been proposed. The Standardized Precipitation Index (SPI) requires only long-term precipitation data and is recommended by the World Meteorological Organization^29^. Its biggest strength lies in the ability to characterize the response time of different usable water sources to precipitation deficits by varying the timescales after a relatively simple calculation^30,31^. However, SPI neglects the effects of evaporation stemming from temperature and other meteorological factors, which leads to misrepresentation of actual drought conditions especially in arid regions^32,33^. The Palmer Drought Severity Index (PDSI) is calculated using a rather complex water-budget system based on historic records of precipitation, temperature and the soil characteristics^34,35^. Therefore, PDSI measures soil moisture deficit and is more suitable for characterizing agricultural drought^36^. The SPEI combines the sensitivity of the PDSI to changes in evaporation demand (caused by temperature fluctuations and trends) with the multitemporal nature of the SPI^32^. It not only accounts for the effect of evaporation on drought, but also characterizes different types of droughts at multiple timescales^37,38^. This makes SPEI more informative in the aspect of actual drought effects over various natural systems and socioeconomic sectors^21,30^.

There are two widely used global SPEI datasets: SPEIbase^39^ and Global Precipitation Climatology Centre Drought Index (GPCC-DI)^40^. The monthly SPEIbase is developed from Climatic Research Unit Time Series datasets with a 0.5° spatial resolution, while monthly GPCC-DI is developed from GPCC precipitation data and Climate Prediction Center’s temperature data with a 1° spatial resolution. The coarse spatial resolution of the two datasets restricts them to be only conducted at the regional or global scale. Meanwhile, the monthly temporal resolution also limits their ability to analyze droughts at sub-seasonal scales or those with a duration less than one month. Although some higher spatial or temporal resolution SPEI datasets have been produced recently^21,33,41,42^, the spatial extents of these datasets are limited to specific regions (e.g., China, pan-African, Central Asia). With the climbing number of sub-seasonal drought researches such as“flash drought”, which require data at a pentad or daily scale, the existing SPEI datasets hardly meet the increasing demand. Therefore, it is urgent to develop a SPEI dataset with daily timescale and global spatial coverage simultaneously.

In such a context, this study developed a global daily SPEI dataset (SPEI-GD) with a 0.25° spatial resolution based on daily precipitation and potential evapotranspiration. The dataset covers the period from 1982 to 2021 and provides five timescales (5, 30, 90, 180 and 360 days). We then evaluated the accuracy of the SPEI-GD against coarser SPEIbase and site-based SPEI, the SPEI-GD was proved to identify more precisely both the spatial extent of drought and the onset and end time of drought. These improvements are critical for monitoring and assessing drought, especially for the accelerating flash drought.

## Methods

### Precipitation

The hourly precipitation data with a 0.25°spatial resolution for the period 1982–2021 was obtained from the ERA5 dataset (https://cds.climate.copernicus.eu/cdsapp#!/dataset/reanalysis-era5-land?tab=form). The hourly precipitation was summed to the daily scale. The ERA5 is the latest reanalysis data from European Center for Medium Weather Forecasting Reanalysis (ERA) and replaces the ERA‐Interim, which has been proved to be highly reliable for investigating climate change^43^. The ERA5 uses more historical observations (especially satellite data) in advanced data assimilation and modelling systems to estimate atmospheric conditions more accurately^44^. The 4D-Var data assimilation technique in cycle 41r2 is applied as well, which can help to explain errors between observation and prediction models, giving users more confidence in analysing atmospheric parameters at different scales of time and space^45^.

### Potential evapotranspiration

The gridded potential evapotranspiration (PET) data developed by Singer *et al*.^46^ for the period 1982–2021 was used (10.5523/bris.qb8ujazzda0s2aykkv0oq0ctp). This PET data was produced based on output from the ERA5-Land reanalysis dataset, over the period from 1981 to present, with hourly temporal resolution and 0.1° spatial resolution. This PET data was calculated via the FAO’s Penman-Monteith (PM) method, which required seven climate variables including zonal and meridional components of wind speed, air and dew point temperature, net solar and net thermal components of radiation, and atmospheric pressure at the Earth’s surface. Compared to the Priestley-Taylor (PT) method, the PM method considers adiabatic sources of energy to drive evaporation. Therefore, Singer’s PET can not only have a broadly similar geographical pattern to the PET products (GLEAM and PT-JPL) based on the PT method, but also more accurately characterize low PET values in northern latitudes (due to low atmospheric energy availability), and high PET values in equatorial region and the Southern Hemisphere^46^. In addition, Sheffield *et al*. have shown that the PM method takes full account of changes in available energy, humidity, and wind speed, while the Thornthwaite method simply takes into account temperature, resulting in an overestimation of PET^35^. To match the precipitation, the spatial resolution of Singer’s PET was resampled to 0.25° based on bilinear method, and the originally hourly data was integrated to daily data.

### SPEIbase

In order to verify the reliability of the SPEI-GD in this study, the SPEIbase for the period 1982–2020 was used (https://digital.csic.es/handle/10261/332007). The SPEIbase is based on monthly precipitation and potential evapotranspiration from the Climatic Research Unit of the University of East Anglia following the FAO-56 Penman-Monteith estimation of potential evapotranspiration^39^. The SPEIbase offers SPEI data at multi-timescales between 1 and 48 months, with a 0.5° spatial resolution and a monthly temporal resolution. To date, it covers the period between January 1901 and December 2020. The SPEIbase has been evaluated and applied by many studies^47–50^. In comparative analysis, in order to match the spatial resolution of SPEIbase, the spatial resolution of new developed SPEI-GD was resampled to 0.5° based on bilinear method.

### Daily and site-based SPEI

Developed by Wang *et al*.^41^ for the period 1982–2018, the daily and site-based SPEI was used to evaluate the performance of SPEI-GD. This data based on multiple factors (daily precipitation, daily average air temperature, daily minimum air temperature, daily maximum air temperature, and sunshine duration) from 1961 to 2018 at 427 meteorological stations across China (10.6084/m9.figshare.12568280). It should be noted that the daily potential evapotranspiration was calculated by the Hargreaves model based on temperature and solar radiation^51,52^. Although air temperature and solar radiation can explain at least 80% of evapotranspiration variability, they also introduce uncertainty into this site-based SPEI data.

### Soil moisture

The SPEI-GD was compared with surface soil moisture (SSM) and root zone soil moisture (RSM) from Global Land-surface Evaporation: the Amsterdam Methodology (GLEAM) version 3.6 dataset (https://www.gleam.eu/#downloads) to further assess its robustness. By this way, daily SSM and RSM data with a spatial resolution of 0.25° during 1982–2021 were obtained. GLEAM is designed to estimate land surface evaporation and root zone soil moisture based on remote sensing observations and reanalysis data^53,54^. The root zone soil moisture is derived from a multilayer water balance driven by precipitation observations and updated with microwave soil moisture estimation. To correct random forcing errors, observations of surface soil moisture were also assimilated into the soil profile.

### Aridity index

Climate regions based on aridity index (AI) were used to further verify the correlation between SPEI-GD and soil moisture. The annual mean aridity index developed by Zomer *et al*.^55^ for the period 1970–2000 with nearly 1 km spatial resolution was applied (10.6084/m9.figshare.7504448.v5). The AI was calculated by the ratio of precipitation to PET, where PET was calculated according to the FAO-56 Penman-Monteith formula. According to the assessment, there is a high correlation between this dataset and the station-based data. AI values are unitless, increasing with more humid condition and decreasing with more arid conditions. Therefore, the climate regions were divided according to the AI values: hyper-arid (AI < 0.03), arid (0.03 ≤ AI < 0.2), semi-arid (0.2 ≤ AI < 0.5), sub-humid (0.5 ≤ AI < 0.65), and humid (AI ≥ 0.65). The spatial resolution of Zomer’s AI was resampled to 0.25° based on bilinear method.

### SPEI calculation

The calculation of SPEI requires the accumulating deficit or surplus (*D*_*i*_) of water balance^40^ at different timescales (5, 30, 90, 180, and 360 days). *D*_*i*_ were calculated by subtracting PET from precipitation using the following equation at a given day *i*:

1 $${D}_{i}={P}_{i}-PE{T}_{i}$$

The obtained *D*_*i*_ were summed at different timescales *D*^*k*^_*j,i*_, which represented a given day *i* and year *j* depending on the chosen timescale *k* (days). The equation is as follow:

2 $$\begin{array}{l}{D}_{j,i}^{k}={\sum }_{l=365(366)-k+i+1}^{365(366)}{D}_{j-1,l}+{\sum }_{l=1}^{i}{D}_{j,l}\quad if\;i\; < \;k\;and\;\\ {D}_{j,i}^{k}={\sum }_{l=i-k+1}^{i}{D}_{j,l}\quad if\;i\;\ge \;k\end{array}$$

where 365(366) represents the number of days in a non-leap or leap year, respectively. The *D*^*k*^_*j,i*_were then normalized into a log-logistic probability distribution, which was recommended to be the best for calculating SPEI^32,56^. The probability density function of log-logistic is as follow:

3 $$f\left(x\right)=\frac{\beta }{\alpha }\left(\frac{x-\gamma }{\alpha }\right){\left[1+\left(\frac{x-\gamma }{\alpha }\right)\right]}^{-2}$$

where the parameters *β*, *γ* and *α* indicate shape, origin and scale, respectively. The probability distribution function of log-logistic is as follow:

4 $$F\left(x\right)={\left[1+{\left(\frac{\alpha }{x-\gamma }\right)}^{\beta }\right]}^{-1}$$

Finally, the SPEI was obtained by standardizing the *F(x)* using the following equation:

5 $$SPEI=W-\frac{{C}_{0}+{C}_{1}W+{C}_{2}{W}^{2}}{1+{d}_{1}W+{d}_{2}{W}^{2}+{d}_{3}{W}^{3}}$$

where *C*_*0*_ = 2.515517, *C*_1_ = 0.802853, *C*_2_ = 0.010328, *d*_1_ = 1.432788, *d*_2_ = 0.189269, and *d*_3_ = 0.001308. The values of *W* were calculated as below:

6 $$W=\sqrt{-2ln\left(P\right)}$$

where *P* = 1 − *F(x)*, when *P* ≤ 0.5. If *P* > 0.5, the *P* is replaced by (1 − *P*) and the sign of SPEI is reversed.

The negative and positive SPEI indicate dry and wet conditions, respectively. The classifications of dry and wet conditions based on SPEI are presented in Table 1, which is similar to the classifications of SPI^57^. Due to low hydroclimatic variability, the SPEI was not reliable over sparsely vegetated and barren areas^39,56^, the two specific land cover types were masked based on the International Geosphere-Biosphere Programme (IGBP) classification of Moderate Resolution Imaging Spectroradiometer (MODIS) landcover product (MCD12C1v061)^58^.

**Table 1:** Classification of dry and wet conditions indicated by SPEI.

SPEI	Climate condition
SPEI ≥ 2.0	Extremely wet
1.5 ≤ SPEI < 2.0	Severely wet
1.0 ≤ SPEI < 1.5	Moderately wet
0.5 < SPEI < 1.0	Mildly wet
−0.5 ≤ SPEI ≤ 0.5	Normal
−1.0 < SPEI < −0.5	Mildly dry
−1.5 < SPEI ≤ −1.0	Moderately dry
−2.0 < SPEI ≤ −1.5	Severely dry
SPEI ≤ −2.0	Extremely dry

## Data Records

The global daily SPEI dataset^59^ (SPEI-GD) at 0.25° spatial resolution from 1982 to 2021 are provided open access via Zenodo, available at 10.5281/zenodo.8060268. This depository includes the five files of the daily SPEI data with five timescales (5, 30, 90, 180, and 360 days). All data are geographical latitude-longitude projection and NetCDF format.

## Technical Validation

To compare the daily SPEI-GD with the monthly SPEIbase, in this study we compared the SPEI of the selected month of SPEIbase with the SPEI of the last day of the corresponding month of SPEI-GD at multi-timescales (30, 90, 180 and 360 days). For example, when comparing with SPEIbase at the 1-month timescale of June 1995, the SPEI at the 181^st^ day (corresponding to the last day of June 1995) of SPEI-GD at 30 days’ timescale would be used. Based on such correspondence, the correlation between SPEI-GD and SPEIbase was further analyzed using the Pearson’s correlation coefficient, and only statistically significant results were accepted and presented. Meanwhile, the Pearson’s correlation was also conducted to reveal the relevance between the site-based SPEI and SPEI-GD over China. Because neither SPEIbase nor site-based SPEI provided SPEI at a timescale shorter than one month, we did not validate SPEI-GD at a 5 days’ timescale. It is worth noting that the SPEI-GD at 5 days’ timescale used the same parameters as the SPEI-GD at other timescales. Soil moisture is an important drought assessment index^28^, and previous studies have found that it has the highest correlation with 6-month SPEI^60^, and this relationship has been applied to verify the accuracy of new SPEI dataset^21,33^. Therefore, the correlation between daily SPEI-GD at 180 days’ timescale and daily soil moisture (SSM and RSM) were evaluated both temporally and spatially.

### Evaluation of the SPEI-GD with monthly SPEIbase

**Fig. 1:**
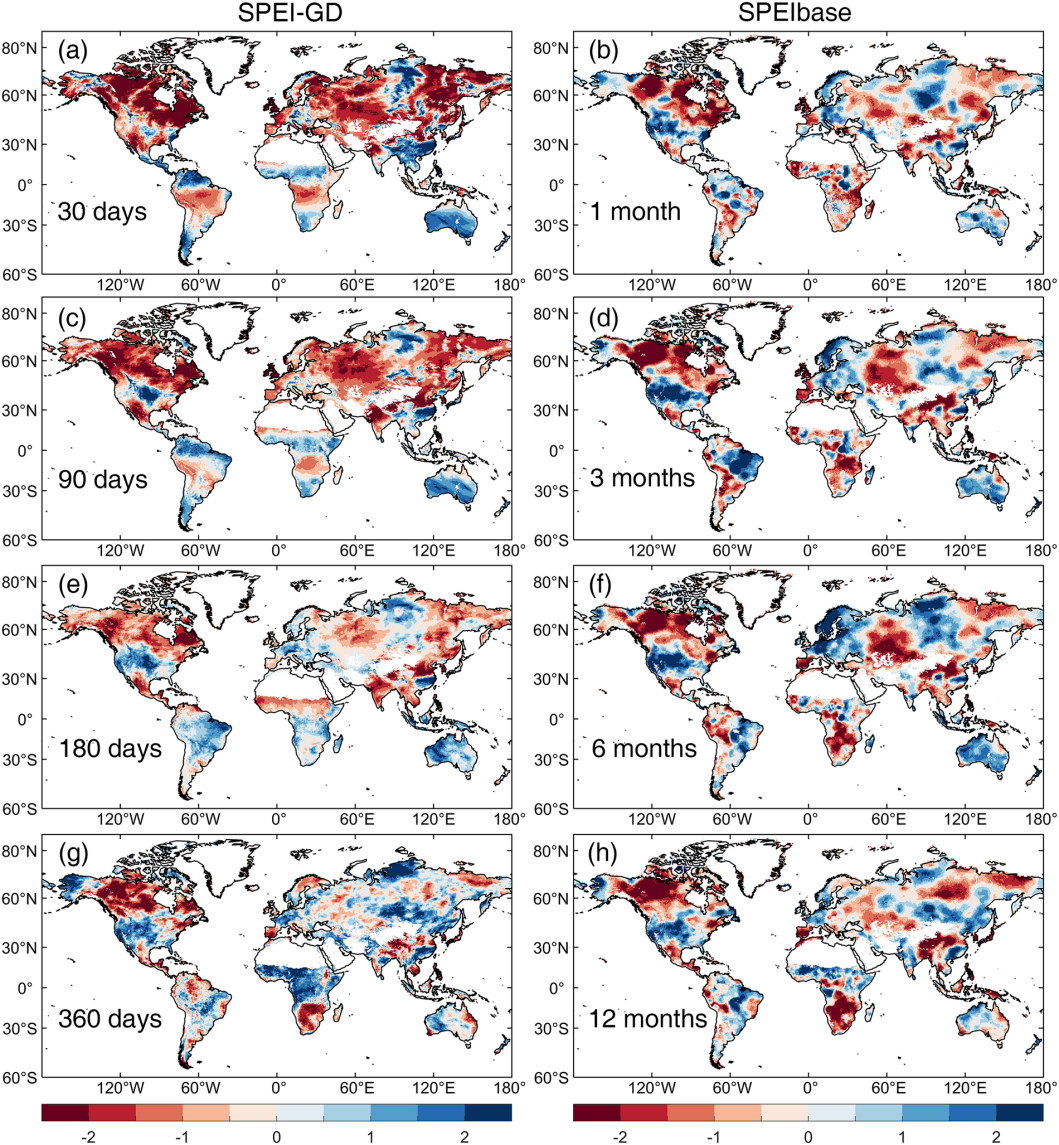
Spatial patterns of multi-scales SPEI in June 1995 based on daily SPEI-GD and monthly SPEIbase. In order to match the temporal scales, the 30, 90, 180 and 360 days of SPEI-GD corresponds to 1, 3, 6 and 12 months of SPEIbase, respectively. The SPEI for the 181^st^ day of SPEI-GD corresponds to that for June of SPEIbase in 1995. The daily SPEI-GD is calculated from ERA5 precipitation and Singer’s PET with a 0.25° spatial resolution, while the monthly SPEIbase is calculated from CRU TS datasets with a 0.5° spatial resolution.

The global pattern of SPEI at an example day (30 June 1995) at multi-timescales (30, 90, 180 and 360 days) obtained from the daily SPEI-GD were shown in Fig. 1 in comparison with SPEI obtained from monthly SPEIbase at the corresponding month (June 1995) and timescales (1, 3, 6 and 12 months). The reasons for choosing June 30 were as follows: on the one hand, the variabilities of precipitation and temperature in June were relatively higher, the differences of SPEI driven by different precipitation and temperature were larger, and thus June was a good time to prove the robustness of SPEI-GD; on the other hand, June 30 is the end of June, the daily timescale (30, 90, 180 and 360 days) of SPEI-GD could exactly correspond to the monthly timescale (1, 3, 6 and 12 months) of SPEIbase. As for 1995, it was just a random year in the time span of the SPEI-GD. The daily SPEI-GD and monthly SPEIbase showed quite similar dry and wet patterns. However, their details differed to some extent, especially in tropical rainforest of the Southern Hemisphere, where the SPEI-GD showed higher SPEI than SPEIbase, implying a lower severity of drought.

Furthermore, the SPEI-GD showed much more spatial details due to higher spatial resolution. Specifically, the spatial dry and wet patterns presented by SPEIbase mostly showed isolated high-value (absolute) centers, while SPEI-GD presented not only high-value centers, but also relatively low values around these centers. In the case of drought assessment, SPEI-GD could identify the spatial extent of the impacts caused by severe drought center, as well as the extent of potential impacts caused by the moderate drought around the center. Meanwhile, the spatial information of the gradient change of drought degree displayed by SPEI-GD could be conducive to the attribution analysis of drought, combined with the spatial change of climatic factors (e.g., precipitation, PET or temperature). These advantages have led to discrepancies between SPEI-GD and SPEIbase in identifying spatial extent of drought, especially across Eurasia. This is because the daily resolution of SPEI-GD showed the spatial extent of both the long-time severe drought and the short-time mild drought, while the monthly resolution of SPEIbase smoothed out the mild drought and showed only the long-time severe drought. As the timescale of SPEI increases, the duration of water deficit gradually escalates as well. For example, in June 1995, northern North America experienced a long-time and extremely severe drought (lasting at least more than a year), while north-central Asia experienced a short drought (lasting about 90 days). This trait can be used to separate meteorological (30 days’ timescale), agricultural (90–180 days’ timescale), and hydrological (360 days’ timescale) droughts^21,41^.

**Fig. 2:**
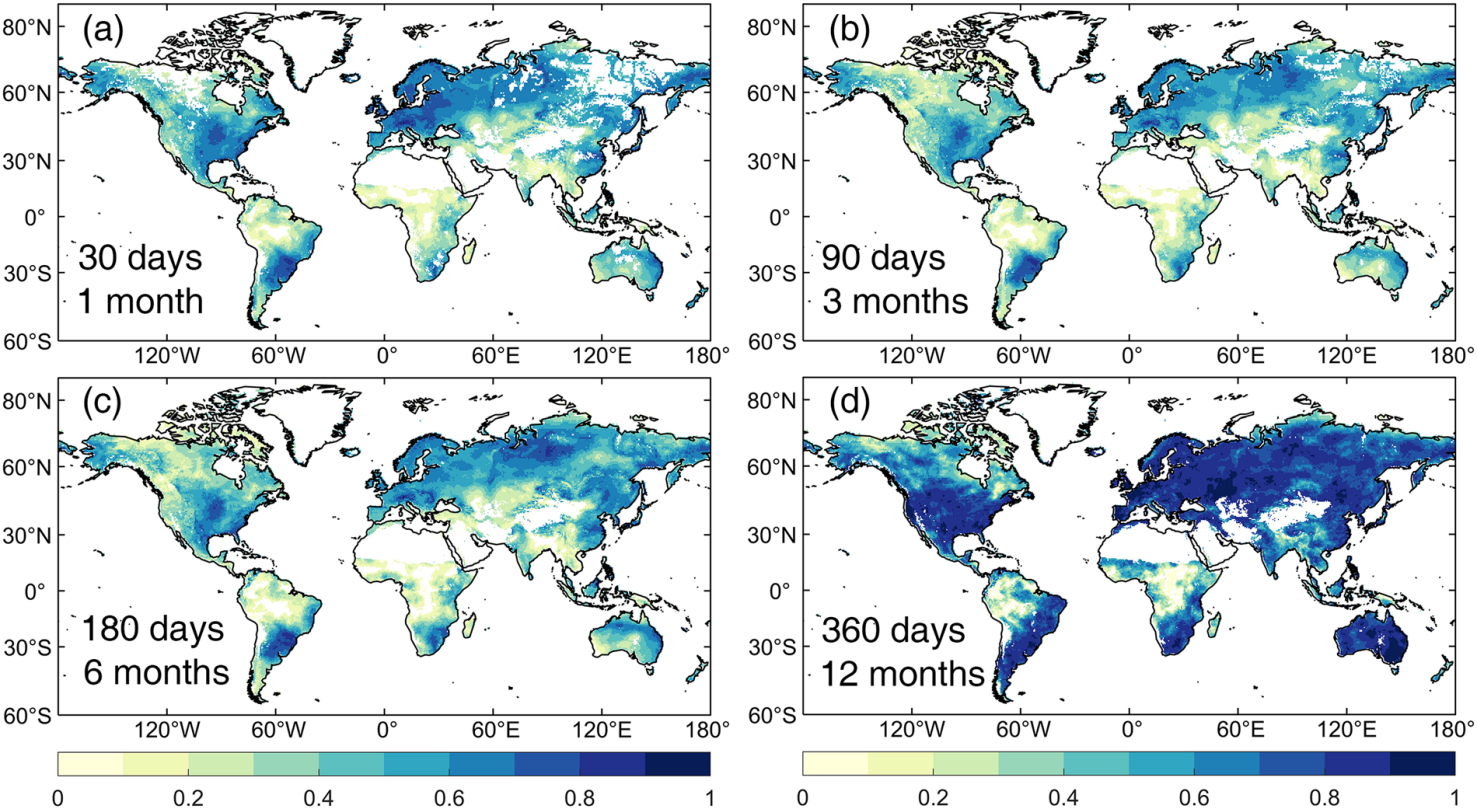
Statistically significant (*p* < 0.05) correlation between SPEI-GD and SPEIbase at different timescales during 1982–2020. The numbers of lower left corner of each subplot indicate the timescale, with days for SPEI-GD, and months for SPEIbase. For spatial matching, the original resolution of 0.25° of SPEI-GD was resampled to 0.5°, consistent with SPEIbase.

To further quantify the difference between SPEI-GD and SPEIbase, the correlation between them was calculated spatially at multi-timescales (Fig. 2). In general, the SPEI-GD and SPEIbase were more consistent at longer timescale. This is because longer timescale not only integrates more water deficits, but also loses more information on water changes, making the two SPEI series smoother and thus more correlated. Furthermore, the correlation existed obviously spatial heterogeneity, with high correlations in America, Europe and China, while low correlations in Amazon, central Africa and central Asia. The input data of SPEIbase relies heavily on meteorological stations, and the number of stations in latter regions is very few, resulting in low accuracy of SPEIbase. In contrast, the newly developed SPEI-GD data in this study is based on reanalysis data, which combines data from multiple sources such as satellites, observation sites and models, largely avoiding the problems caused by the lack of input data.

**Fig. 3:**
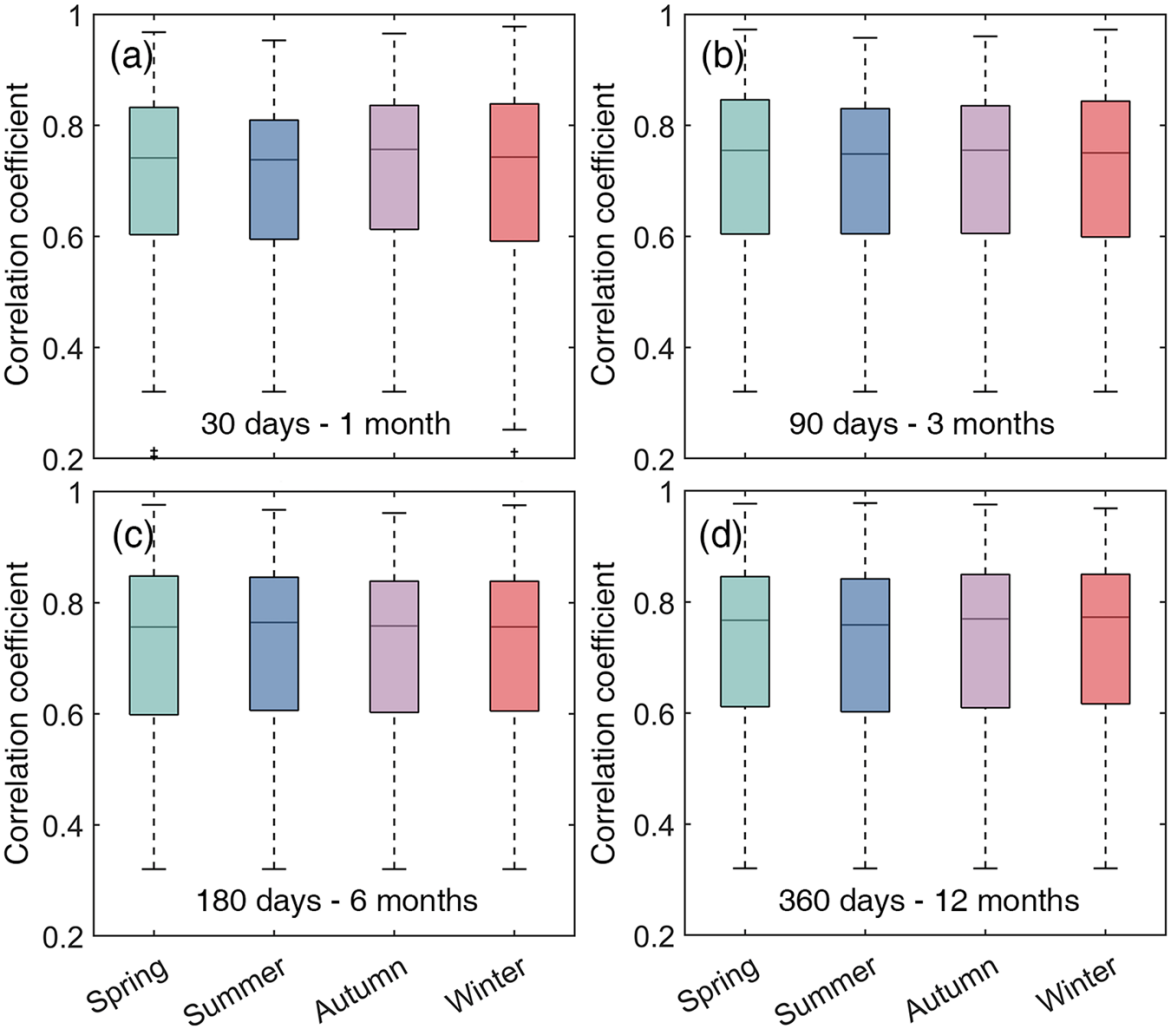
Statistically significant (*p* < 0.05) correlation between SPEI-GD and SPEIbase at different timescales in different seasons during 1982–2018. The numbers of lower middle corner of each subplot indicate the timescale, with days for SPEI-GD, and months for SPEIbase.

Additionally, the correlations between SPEI-GD and SPEIbase at different timescales in different seasons were compared over the entire period (Fig. 3). The correlation coefficients in each season were averaged in the corresponding months in each hemisphere. For example, summer correlation coefficients were averaged from the monthly correlation coefficients for June to August in the Northern Hemisphere and December to February in the Southern Hemisphere. In general, the SPEI-GD and SPEIbase matched well with each other in all seasons, with median correlation coefficients greater than 0.70. Lower correlation coefficients were mainly found in the Amazon, central Africa and northern North America (Figs. S1–S4 in the Supplementary Information). Considering that SPEIbase was already widely used in drought monitoring in different seasons, the strong correlation between SPEI-GD and SPEIbase confirmed that SPEI-GD would also be highly reliable in drought monitoring in different seasons. The median correlation coefficients had a slight tendency to increase as the timescale increased. This is because the larger timescale smoothed out short-term fluctuations in the SPEI series. The lower correlation coefficients in summer may be attributed to the greater variability of temperature and precipitation, when the ERA5 and CRU data used to calculate SPEI-GD and SPEIbase respectively, were biased.

### Evaluation of the SPEI-GD with daily site-based SPEI

**Fig. 4:**
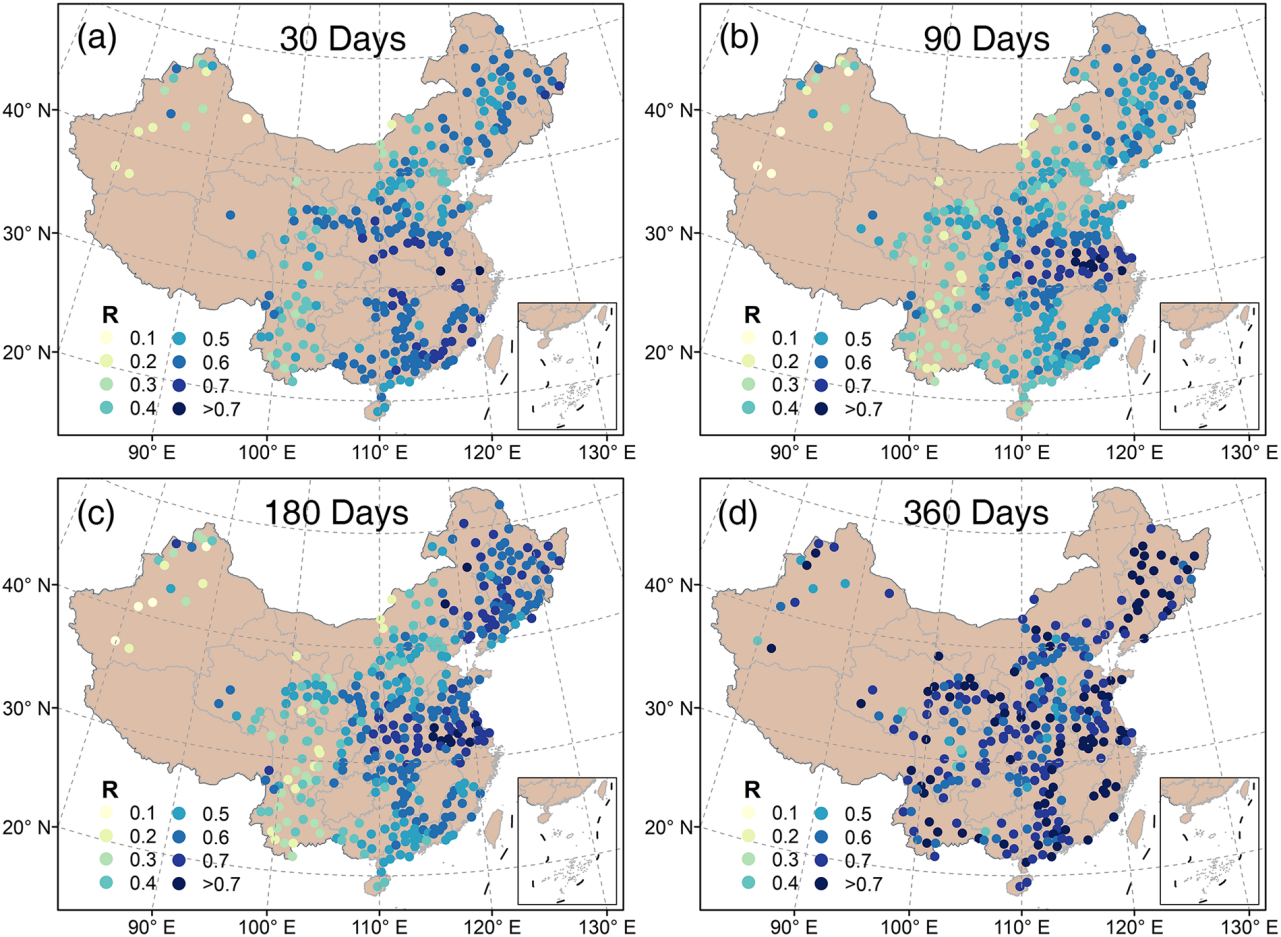
Statistically significant (*p* < 0.05) correlation between SPEI-GD and site-based SPEI at different timescales during 1982–2018. The numbers of upper center of each subplot indicate the timescale. SPEI-GD and site-based SPEI are both daily data.

Data products based on the inversion of site observations are considered to be the most reliable. In this study we further calculated the correlation between SPEI-GD and site-based SPEI, and specifically, the daily SPEI-GD of the raster at the station location of site-based SPEI was used (Fig. 4). In general, positive correlations with mostly larger than 0.5 (*p* < 0.05) between SPEI-GD and site-based SPEI were found at each timescale (30, 90, 180 and 360 days), and especially, for 360 days. Considering the sample size of 13880 (38 years with 365/366 days for each year) involved in the correlation analysis, a correlation coefficient of 0.5 or more indicated that the SPEI-GD developed in this study had a very high accuracy against site-based SPEI. However, the relatively low correlation coefficients in the arid regions of northwest China and drought-prone southwest China^61^ may result from the low hydroclimatic variability, and the large uncertainty in the SPEI series^39,56^.

### Comparison against surface and root zone soil moisture

**Fig. 5:**
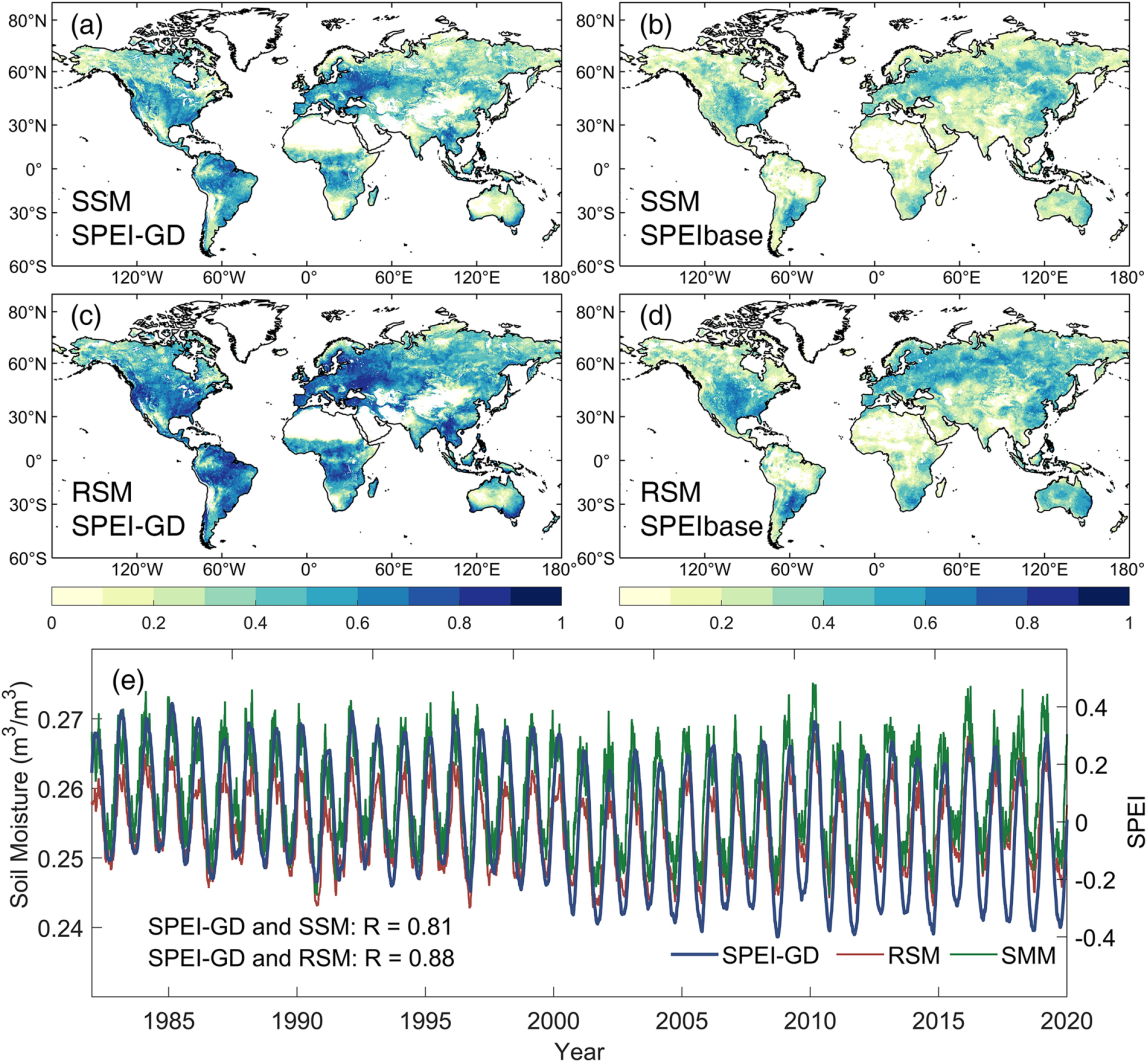
Statistically significant (*p* < 0.05) correlation between daily SPEI-GD, SPEIbase and soil moisture at the timescales of 180 days and 6 months during 1982–2020. (**a,b**) Surface soil moisture (SSM). (**c,d**) Root zone soil moisture (RSM). (**e**) The time series of global mean SSM, RSM and SPEI-GD, where R indicates the correlation coefficient.

The SPEI-GD and SPEIbase were also compared with surface and root zone soil moisture at the timescales of 180 days and 6 months during 1982–2020. As shown in Fig. 5, SPEI-GD showed stronger positive correlations with SSM and RSM than SPEIbase globally, especially in Amazon and central Africa. Given the large uncertainty of SPEIbase in these regions as mentioned above, the results presented here suggested a higher accuracy of SPEI-GD than SPEIbase. In addition, both SPEI-GD and SPEIbase showed relatively higher correlations with RSM than SSM. This is mainly due to the fact that SSM is more susceptible to non-meteorological factors such as vegetation activities and human activities than RSM, and thus has a relatively lower correlation with the meteorological drought index of SPEI^62^. This interpretation can be further verified from Fig. 5e, which calculated the daily global mean of SSM, RSM, and SPEI-GD. The amplitude of SSM was relatively lower than RSM, and the time series were more stable. The correlation coefficient between global means of SPEI-GD and SSM was as high as 0.81 with 0.88 for that between SPEI-GD and RSM, further indicating the high accuracy of SPEI-GD.

**Fig. 6:**
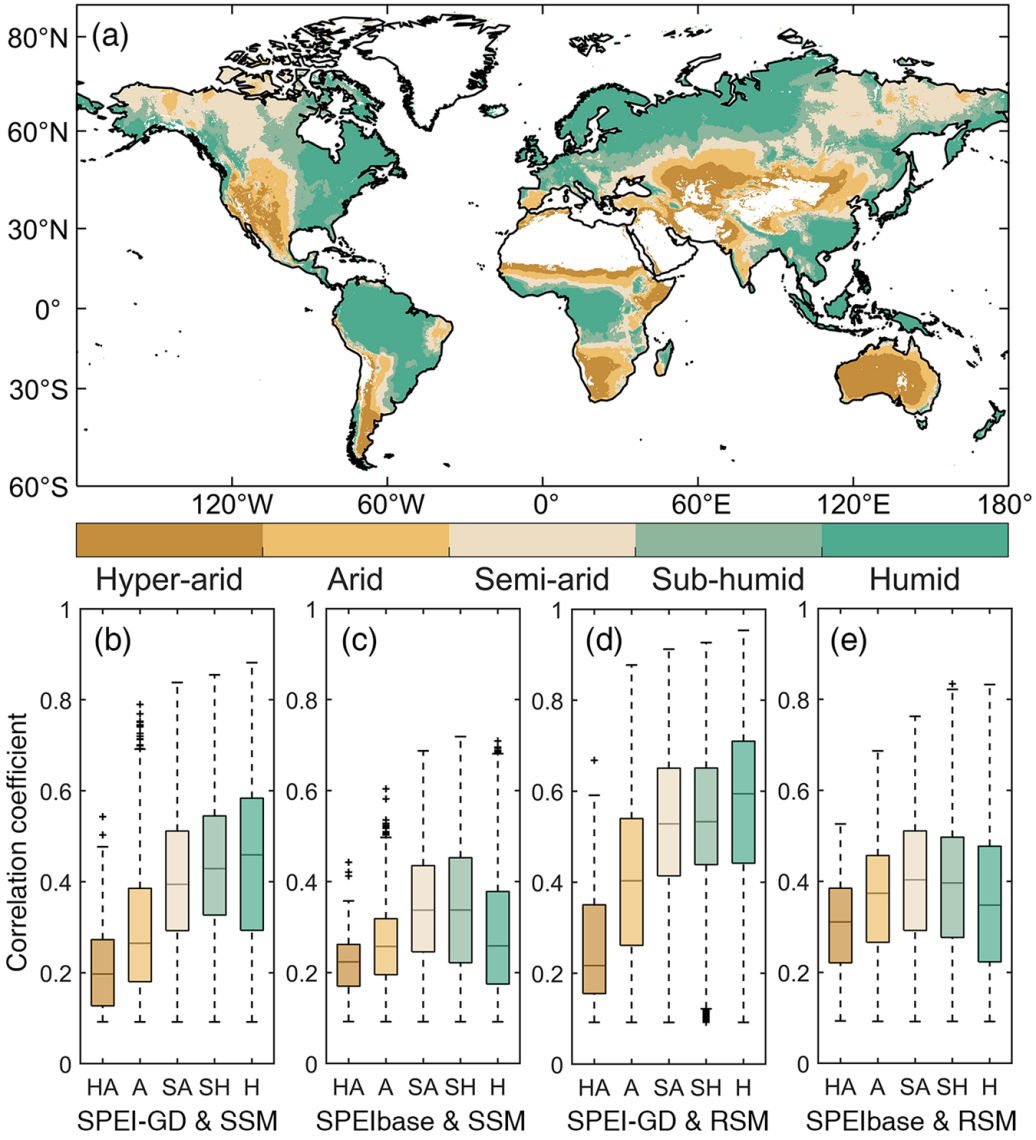
Statistically significant (*p* < 0.05) correlation between SPEI-GD, SPEIbase and soil moisture at the timescales of 180 days and 6 months during 1982–2020 in different climate regions. (**a**) Spatial pattern of global climate regions classified based on AI. (**b**) Correlation between SPEI-GD and SSM. (**c**) Correlation between SPEIbase and SSM. (**d**) Correlation between SPEI-GD and RSM. (**e**) Correlation between SPEIbase and RSM.

In addition, the correlation between SPEI-GD, SPEIbase and soil moisture in different climate regions were calculated (Fig. 6). Overall, no matter SSM or RSM, their correlation with SPEI-GD were higher than that with SPEIbase in almost all climate regions, except hyper-arid region. Compared with SPEIbase, the increase of correlation coefficient of SPEI-GD with soil moisture was particularly significant in humid and sub-humid regions. In details, in the sub-humid region, the median correlation coefficient with RSM (SSM) increased from 0.40 (0.34) to 0.53 (0.43); in the humid region, the median correlation coefficient with RSM (SMM) increased from 0.35 (0.26) to 0.60 (0.46). The correlation between SPEI-GD and soil moisture gradually increased with decreased dryness, which was expected that higher water availability in wetter regions leaded to slower soil moisture consumption and thus more correlated with longer timescales SPEI. In contrast, the median correlation coefficient between SPEIbase and soil moisture was lower in sub-humid and humid region than in semi-arid region, possibly because the CRU data driving SPEIbase had a larger error in sub-humid and humid regions.

### Summary

Based on ERA5 daily precipitation and Singer’s daily potential evapotranspiration, the global daily SPEI dataset (SPEI-GD) was produced. The SPEI-GD dataset covers the complete daily series of 40 years from 1982 to 2021 with a spatial resolution of 0.25°. The SPEI-GD provides multi-timescales accumulated SPEI, including 5, 30, 90, 180 and 360 days. We hope this new dataset can reduce the cost of time for researchers and avoid duplication of effort. Compared with existing SPEI datasets (SPEIbase and site-based SPEI for China), SPEI-GD not only has higher temporal and spatial resolution, and wider spatial extent, but also has a higher correlation with surface and root zone soil moisture from GLEAM, especially in areas where site observations are missing, such as Amazon and central Africa tropical rainforests. These results indicate our new dataset improves the spatial and temporal resolution of the available SPEI data while enhancing the simulation accuracy.

These improvements can better support global or regional drought monitoring and response at a daily scale, such as quantifying the characteristics of sub-seasonal droughts^63,64^, including accurately extracting the onset and end days of various droughts instead of the onset and end months, as well as the number of drought duration days. Based on daily SPEI-GD data, the onset or end dates of the drought could be determined based on SPEI below or above a certain threshold. For example, a specific day with the first SPEI < −1 indicates the onset date of a moderate drought, and the followed day with SPEI > −0.5 indicates the end date of the drought. The number of days of drought duration could be determined by the difference between the onset date and end date. Through the analysis of these characteristics at daily scale, the evolution process of drought could be accurately clarified.

Flash droughts are typically sub-seasonal droughts characterized by rapid onset that can develop into severe droughts within a few weeks^28^. The SPEI has been proved to be a reliable and robust metric to identify and quantify flash drought^65–67^, and a flash drought is defined to have: (1) a minimum length of 4 weeks in the development phase; (2) a ΔSPEI equals to or < −2 z-units; and (3) a final SPEI equals to or < −1.28 z-units. Due to the lack of daily SPEI, SPEI at 1-month timescale or weekly time resolution was used in previous studies, resulting in the characterizing of flash droughts (duration, and timing of onset and end) only at a weekly time resolution. Our SPEI-GD data with daily time resolution can further improve the temporal accuracy of flash drought characteristics (e.g., which day its onset or end is, and how many days it lasted).

There are also uncertainties that need to be explored in future works. Firstly, in SPEI calculation, we used the widely accepted log-logical probability distribution to fit the deficit or surplus of the water balance, which needed to be further compared with other fitting functions (Normal, Pearson type III and Generalized Extreme Value). Secondly, we mainly used the reanalysis data of ERA5 as input data. Future work could integrate observational data to improve the robustness of SPEI-GD, such as daily precipitation data from Global Precipitation Climatology Centre (GPCC), and daily temperature data from Climate Prediction Center (CPC). Lastly, we proposed the advantage of daily SPEI-GD in identifying flash drought characteristics, but the associated thresholds (e.g., the minimum lasted days in the development phase) needed to be further determined.

### Supplementary information

Supplementary Information

## Data Availability

Since the newly developed SPEI-GD dataset has a high temporal and spatial resolution (the amount of data in the intermediate process is about 2 000 GB), we ran codes on MATLAB and R programming Language, utilizing parallel computing tools and chunked computation algorithms to solve the problem of limited computer memory and long computation time. The code files are available at https://github.com/XuebangLiu/SPEI-GD.
