# Supplementary material for: The first global multi-timescale daily SPEI dataset from 1982 to 2021
Source: Sci Data. 2024 Feb 21;11:223. doi: 10.1038/s41597-024-03047-z (PMC10881487; doi:10.1038/s41597-024-03047-z)
Supplement: Supplementary file 1 — Supplementary Information [file 41597_2024_3047_MOESM1_ESM.pdf]

Supplementary Information

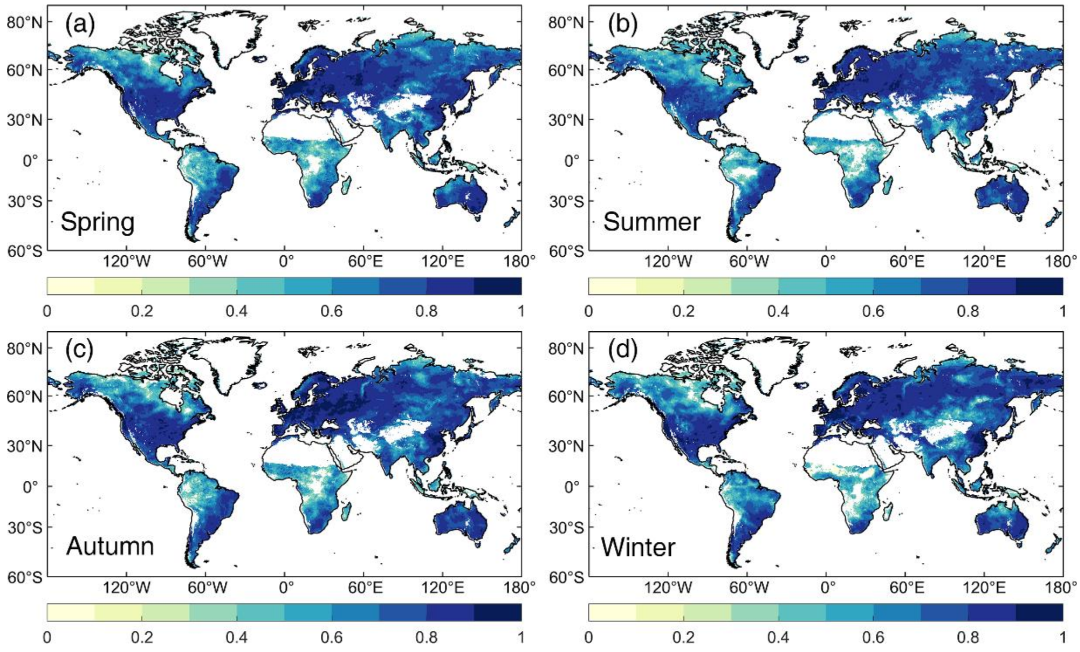

Fig. S1 Statistically significant (p<0.05) correlation between SPEI-GD and SPEIbase at 30days' or 1-month' timescale in different seasons during 1982-2020.

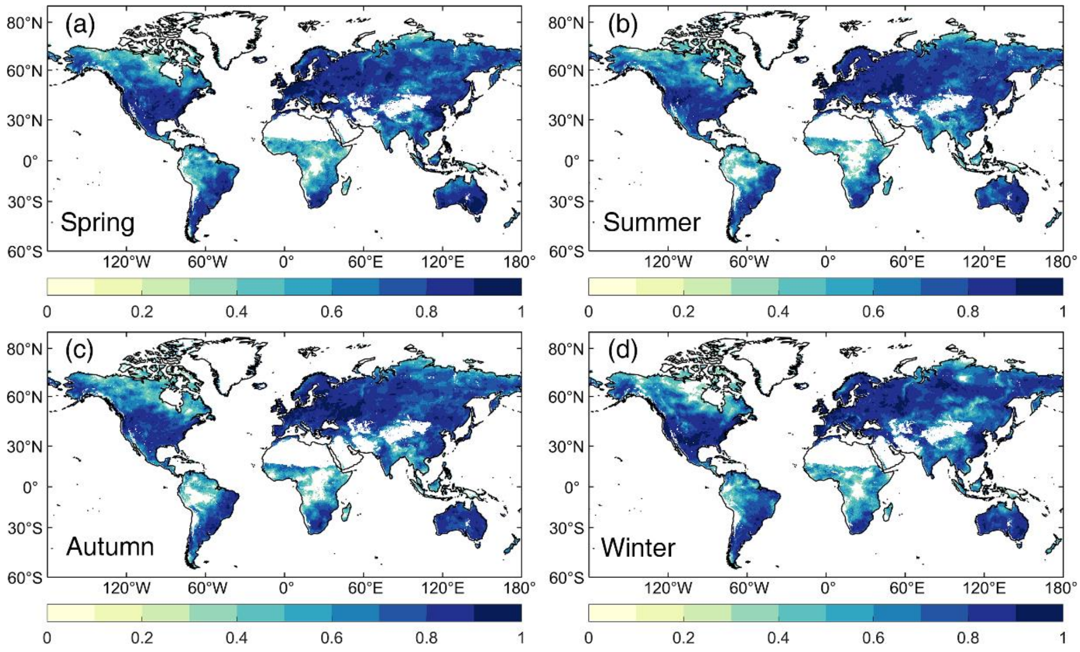

Fig. S2 Statistically significant (p<0.05) correlation between SPEI-GD and SPEIbase at 90days' or 3-months' timescale in different seasons during 1982-2020.

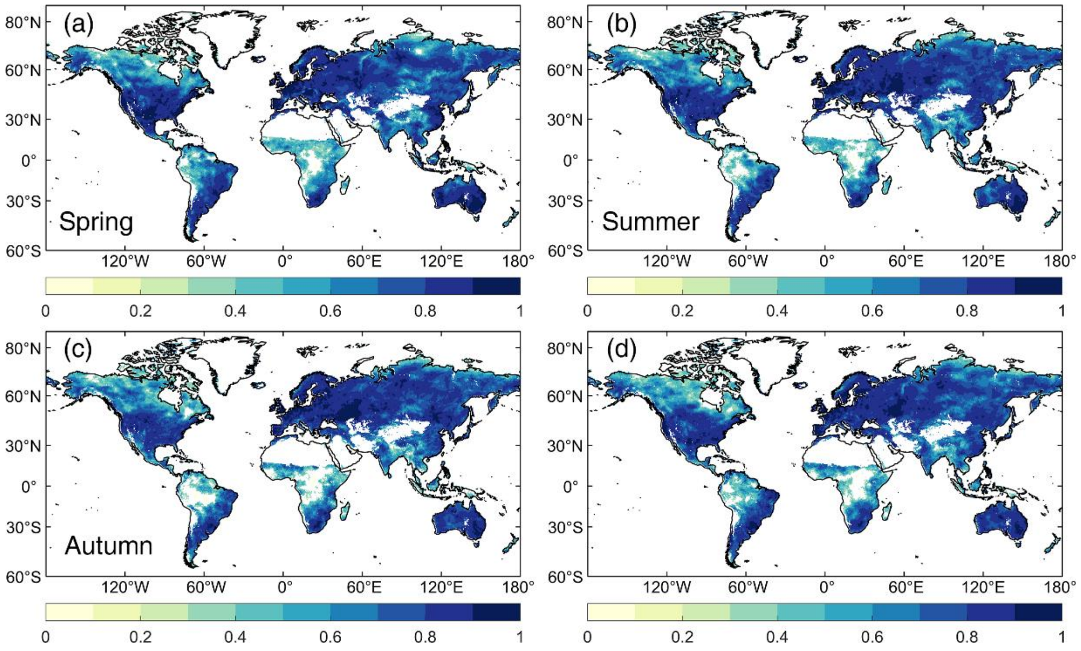

Fig. S3 Statistically significant ($p<0.05$) correlation between SPEI-GD and SPEIbase at 180days' or 6-months' timescale in different seasons during 1982-2020.

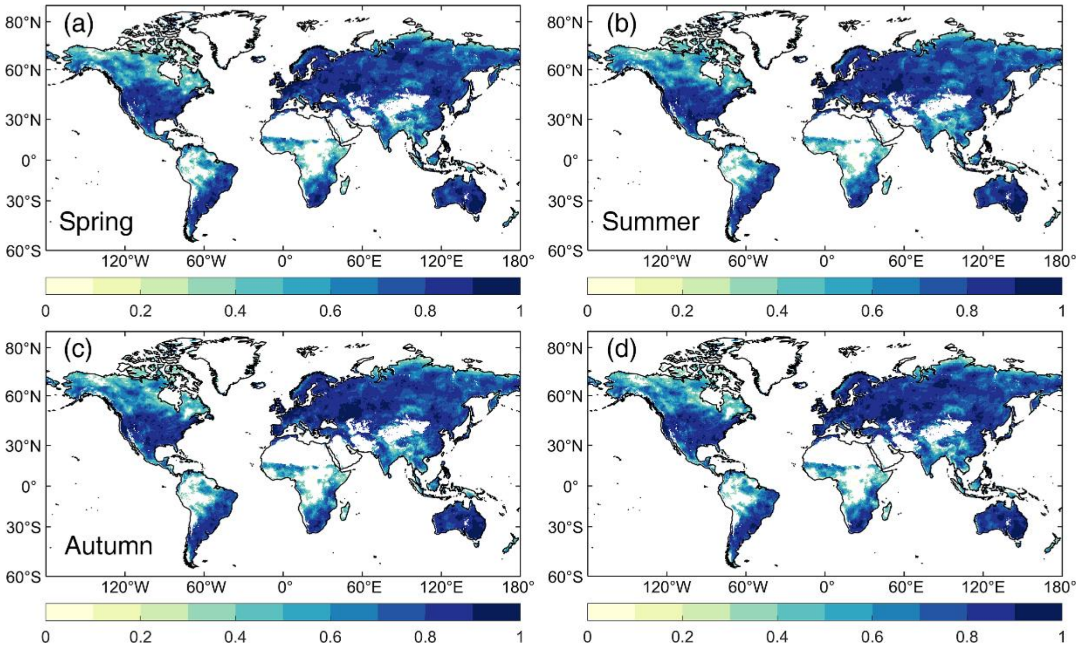

Fig. S4 Statistically significant ($p<0.05$) correlation between SPEI-GD and SPEIbase at 360days' or 12-months' timescale in different seasons during 1982-2020.
